# Activation of Lymphocytes Induced by Bronchial Epithelial Cells with Prolonged RSV Infection

**DOI:** 10.1371/journal.pone.0027113

**Published:** 2011-12-28

**Authors:** Ling Qin, Cheng-ping Hu, Jun-tao Feng, Qian Xia

**Affiliations:** Respiratory Department, Xiangya Hospital, Central South University, Changsha, Hunan, China; University of Liverpool, United Kingdom

## Abstract

Respiratory syncytial virus (RSV) preferentially infects airway epithelial cells,which might be responsible for susceptibility to asthma; however, the underlying mechanism is not clear. This study determined the activation of lymphocytes and drift of helper T (Th) subsets induced by RSV-infected human bronchial epithelial cells (HBECs) *in vitro*. HBECs had prolonged infection with RSV, and lymphocytes isolated from human peripheral blood were co-cultured with RSV-infected HBECs. Four groups were established, as follows: lymphocytes (group L); lymphocytes infected with RSV (group RL); co-culture of lymphocytes with non-infected HBECs (group HL); and co-culture of lymphocytes with infected HBECs (group HRL). After co-culture with HBECs for 24 hours, lymphocytes were collected and the following were determined in the 4 groups: cell cycle status; apoptosis rate; and concentrations of IL-4, IFN-γ, and IL-17 in the supernatants. Cell cycle analysis for lymphocytes showed a significant increase in S phase cells, a decrease in G1 phase cells, and a higher apoptosis rate in group HRL compared with the other three groups. In group HRL, the levels of IL-4, IFN-γ, and IL-17 in supernatants were also higher than the other three groups. For further study, lymphocytes were individually treated with supernatants from non-infected and RSV-infected HBECs for 24 h. We showed that supernatants from RSV-infected HBECs induced the differentiation of Th2 and Th17 subsets, and suppressed the differentiation of Treg subsets. Our results showed that HBECs with prolonged RSV infection can induce lymphocyte proliferation and apoptosis, and enhance the release of cytokines by lymphocytes. Moreover, subset drift might be caused by RSV-infected HBECs.

## Introduction

Respiratory syncytial virus (RSV) is an important respiratory pathogen that produces an annual worldwide epidemic of respiratory illnesses primarily occurring in infants, but also in adults [1]. However, the impact of RSV in the clinic reaches beyond infections, as it has been suggested that infection with RSV may cause a predisposition to the development of asthma [2]. This conclusion stems from the fact that 40%–50% of patients hospitalized with RSV infections subsequently have persistent wheezing [3], [4], and RSV infections have been shown to exacerbate asthma in both children and adults [5], [6]. In our previous studies, airway hyperactivity and related pathologic changes have been reported in RSV-infected animals [7]; however, the mechanisms underlying RSV-induced asthma are incompletely understood.

The mechanisms underlying asthma which are considered important are classically characterized by immune activation and immune imbalance [8]. When exogenous antigen is encountered, antigen presenting cells (APCs) induce T lymphocytes to proliferate and differentiate with the prominent expression of cytokines, which are mainly released by Th cells. In response to antigen stimulation, naive CD4^+^T cells differentiate into different subsets of Th cells that are classified based on distinct cytokine profiles and immune regulatory functions [9], [10]. Th1 cells produce IL-2 and IFN-γ, and play an important role in cell-mediated immune responses against intracellular pathogens. Th2 cells produce IL-4, IL-5, and IL-10, and Th17 cells produce IL-17. Th2 and Th17 subsets are both involved in humoral immunity and allergic responses, such as asthma [11]. Another Th subset, regulatory T (Treg) cells, which are characterized by the *Foxp3* gene, suppress the immune system to prevent overactive responses and inflammation [12], [13].

In recent years, it was well-accepted that bronchial epithelial cells form not only a physical barrier to the external environment, but also contribute to the earliest anti-viral immune responses against foreign antigens. Epithelial cells play an important role in immunologic derangement of the respiratory system. The immune response of epithelia to infection and antigen exposure involves presenting antigens to lymphocytes and releasing chemokines and cytokines into the submucosa, which initiates a local inflammatory reaction [14]. Airway epithelial cells are closely related to asthma, and damage of airway epithelial structure and function may result in susceptibility to asthma, which could be a priming process in asthma [15]. Respiratory epithelial cells are the first and primary target of RSV [16]. Therefore, we hypothesize that bronchial epithelial cells, which are infected with RSV, have an important regulatory effect on immune activation by presenting antigen signals and releasing inflammatory factors. The aim of this study was to determine the level of immune activation and imbalance of lymphocytes *in vitro* when stimulated by RSV-infected human bronchial epithelial cells (HBECs).

## Materials and Methods

### Cell culture

The HBEC line was a gift from the Physiology Department of Central South University (Changsha, Hunan, China) and maintained in complete medium (DMEM containing 10% FBS, 2 mM glutamine, 4500 mg/L of D-glucose, streptomycin [100 U/ml], and penicillin [100 U/ml]). The cells were incubated at 37°C and 5% CO_2_, and used in experiments during the 62^nd^–73^rd^ passages.

### Preparation of RSV

RSV (Long strain/A2 type) was obtained from Guangzhou Medical College (Guangzhou, Guangdong, China) and propagated in a human cervical cancer cell line (Hela cells). Hela cells were purchased from Cell Center of Central South University and cultured in complete medium. Confluent monolayers of Hela cells were infected with RSV for 3 h. The monolayers were washed, overlaid with maintenance media (DMEM containing 2% FBS), and incubated at 37°C in 5% CO_2_ until the cytopathic effects reached 80%. Thereafter, the cells were repeatedly frozen and thawed to facilitate rupture. Next, the supernatants were harvested and cellular debris was removed by centrifugation (200 g for 10 min). The RSV viral suspension was stored at −80°C.

### RSV infection

Confluent monolayer cultures of HBECs were infected with RSV at a multiplicity of infection (MOI) of 0.0001 pfu/cell. For comparison, the dose of RSV to induce an acute cytopathic effect in 50% of the cells (TCID50) is 1.4×10^6^ pfu/mL. A 1-mL viral suspension was added to the cells for 3 h, then removed by a gentle wash with culture medium, followed by addition of maintenance media. The infected cells were then incubated for growth and passage continuously. Thus, we designated the passages of cells as “HBECs with prolonged RSV infection”. Non-infected HBECs were used as a control.

Non-infected HBECs and the RSV prolonged infection model were plated at 2×10^6^ cells/well in a 6-well culture plate and incubated at 37°C in 5% CO_2_. After 12 h, the cells were washed with PBS and cultured in 2 mL of serum-free medium in each well for 24 h. The supernatants were collected and stored at −70°C for further use.

### RSV infection efficiency of HBECs

The cytopathic effects in RSV-infected HBECs were observed under a phase contrast microscope. Using RSV F protein monoclonal antibody (Santa Cruz Biotechnology, Inc., Santa Cruz, CA, USA) as a primary antibody, FITC-conjugated virus particles and efficiency of infection were tested by immunofluorescence (efficiency of infection = [number of positive cells/number of all cells in same visual field] ×100). The cellular ultrastructure and subcellular localization of the virus were observed under electron microscope, as previously described [17].

### Separation of peripheral blood lymphocytes

Heparinized whole blood was collected from healthy adult volunteers. Peripheral blood mononuclear cells (PBMCs) were isolated by Ficoll-Conray (Haoyang Company, Tianjin, China) density gradient centrifugation [18]. Then, PBMCs were incubated in complete medium at 37°C in 5% CO_2_ for 2 h until the monocytes adhered to the bottom of culture flasks. The lymphocytes suspended in medium were isolated by centrifugation. After a viable count with 0.4% trypan blue dye, the density of viable lymphocytes was adjusted to 2×10^6^cells/mL in serum-free medium.

### Co-culture of HBECs and lymphocytes

The HBECs were adherent and the lymphocytes were in suspension. In co-culture experiments, HBECs were located at the bottom of the culture plate and the lymphocytes were suspended in culture medium. Non-infected and prolonged RSV-infected HBECs were plated in 6-well culture plates with 2×10^6^ cells/well. After HBECs adhered to the bottom of the culture plate for 12 h, lymphocytes were added to the HBECs in a 1∶1 ratio so that HBECs and lymphocytes were cultured in the same well.

A 2-mL lymphocyte suspension was added to 6-well plates; the concentration was 2×10^6^ cells/mL. Lymphocytes were then divided into 4 groups, as follows: lymphocytes (group L); lymphocytes infected with RSV (MOI = 0.0001; group RL); co-culture of lymphocytes and non-infected HBECs (group HL); and co-culture of lymphocytes and RSV-infected HBECs (group HRL). After incubation at 37°C in 5% CO_2_ for 24 h, the lymphocytes and supernatants were collected separately.

### Flow cytometric analysis of lymphocyte cell cycle and apoptosis

The collected lymphocytes were gently thrice-washed with PBS, then fixed in 2 ml of 70% cold ethanol at 4°C for at least 18 h. Then, the lymphocytes were twice-washed with PBS and re-suspended in 0.5 ml of propidium iodide (PI)/RNaseA solution. The cell mixture was incubated in dark at room temperature for 10 min. The cell cycle phase and apoptosis of the stained cells were analyzed using flow cytometry (BD Science, USA). Apoptotic cells were indicated by the hypodiploid peaks.

### Cytokine measurement

Human IL-4, IFN-γ and IL-17 concentrations in co-cultured supernatants were determined using ELISA kits from R&D Systems (USA).

### Treatment of lymphocytes by supernatants from HBECs with prolonged RSV infection

The secretory pattern of bronchial epithelial cells (BECs) changes after RSV infection. Thus, RSV-infected BECs can over-secrete a range of cytokines. To determine the regulatory effect of factors secreted by RSV-infected HBECs on Th subset differentiation, the isolated lymphocytes were plated in 6-well culture plates at a density of 4×10^6^cells/well and incubated in 2 ml of the following media: complete medium; supernatants from normal HBECs; and supernatants from RSV-infected HBECs. Phytohemagglutinin (PHA-P, 20 µL, 10 mg/ml; Sigma, St. Louis, MO, USA) was added to each well to stimulate non-specific lymphocyte mitosis. After incubation at 37°C in 5% CO_2_ for 24 h, lymphocytes from the 3 groups were collected and analyzed by fluorescent staining and flow cytometry to test Th subset differentiation.

### Fluorescent staining and flow cytometry analysis

PE anti-human IL-4, PE anti-human IL-17, PE anti-human CD25, and FITC anti-human Foxp3 antibodies were obtained from eBiosicence (USA) and used as staining reagents. For intracellular staining of IL-4 and IL-17, lymphocytes were pre-conditioned in the presence of 2 µL of monensin (×1000; Biolengend, USA) for 6 h, which can inhibit secretion of newly produced cytokines. Then, the cells were fixed with 4% paraformaldehyde for 10 min at room temperature and permeabilized in permeabilizing solution (eBioscience). After blocking with 3% BSA for 15 min, cells were stained with appropriate reagents on ice for 45 min. The surface molecule staining of CD25 was prior to intracellular staining of Foxp3. The Foxp3 staining procedure was the same as the intracellular staining of IL-4 and IL-17. In multi-color flow cytometry analysis, electronic compensation was done using cell mixtures of positive and negative cell populations in each fluorescence emission.

### Statistical analysis

Data are presented as the mean±SEM. Statistical analysis of 2-way ANOVA, followed by *post hoc* testing was used to analyse the cell cycle, apoptosis, and release of cytokines from lymphocytes in the co-culture system. Univariate ANOVA, followed by Dunnett's t-test, was used to analyse Th subset distribution after treatment with supernatants from HBECs. Two-tailed and paired tests were used throughout the whole statistic. A P value<0.05 was considered statistically significant. Statistics were calculated by SPSS/Windows version 13.0 software.

### Ethics Statement

Peripheral blood was withdrawn from healthy adult volunteers. Before the experiments, we obtained approval for our study from the Ethics Committee of Xiangya Hospital. In the medical examination center of Xiangya Hospital, participants were recruited and human experimentation was conducted. We obtained written informed consent from all participants involved in our study.

## Results

### The cytopathic effects observed with phase contrast microscopy

**Figure 1:**
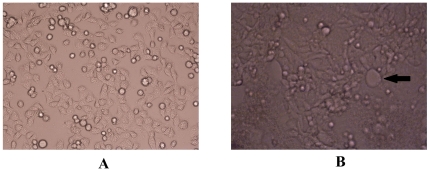
The cytopathic effects in HBECs after infection with RSV were observed under phase contrast microscopy. A. normal HBECs; B. The classical pathologic changes of RSV-infected cells: enlarged fusion cell (×100). In this vision, variant shape, shrinkage, and enhanced refraction were present in cells, as well as enlarged fusion cells and intracytoplasmic eosinophilic inclusion, which were considered to be proof of RSV infection. A fusion cell is indicated by arrows.

In this study we established a prolonged RSV infection model using HBECs. We defined the primary infected generation as passage zero, the passage of which was the first passage, and so on. In the interval after infection with RSV, the cells maintained proliferation and other functions, and could still grow and passage continuously. After several passages (usually the 6^th^–7^th^ passage of RSV-infected HBECs), fusion cells were formed, the rate of proliferation became gradually slower, and apoptosis or shedding began to occur. The period from the first infected passage to the last passage, at which time apoptosis occurred, was designated as the prolonged infection period. The 3^rd^–5^th^ passages of RSV-infected cells showed active proliferative capacity and biological function. Thus, we chose the cells in these stages for the next investigation. No pathologic changes were observed in control cells (Figure 1A). In RSV-infected HBECs, according to the appearance of enlarged fusion cells, the prolonged infection period could be further divided into two stages (early and late infection). In the early infection stage, the cells proliferated actively, but in the late infection stage, the shape of the cells were abnormal, with shrinkage and enhanced refraction, as well as enlarged fusion cells, which were considered to be characteristics of RSV infection (Figure 1B) [19].

### RSV-infected HBECs examined by immunofluorescence

**Figure 2:**
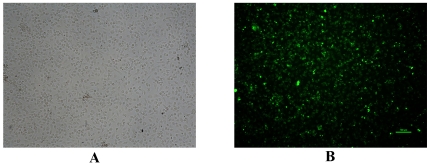
The model of RSV-infected HBECs was examined by immunofluorescence. A. Infected cells can be seen under phase contrast microscopy (×40); B. Infected cells conjugated with FITC can be seen under fluorescence microscopy (×40). Bright yellow green particles of various sizes were distributed within cytoplasm, which suggested the cells were positive for RSV infection and RSV infected model was constructed successfully.

The 3^rd^–5^th^ generations of infected cells (the same cell models were used in subsequent experiments) were obtained for immunofluorescent staining. Under phase contrast microscopy, the infected cells had regular shapes and grew actively (Figure 2A). Under fluorescence microscopy, virus particles of various sizes were conjugated with FITC, which were indicated by bright yellow green fluorescence distributed within the cytoplasm, suggesting that the RSV-infected model was constructed successfully (Figure 2B). The measured efficiency of RSV infection was 31.58%±21.03% (n = 16). There was no fluorescence distributed in control cells which were not infected with RSV.

### Morphologic changes of RSV-infected HBECs under electron microscopy

**Figure 3:**
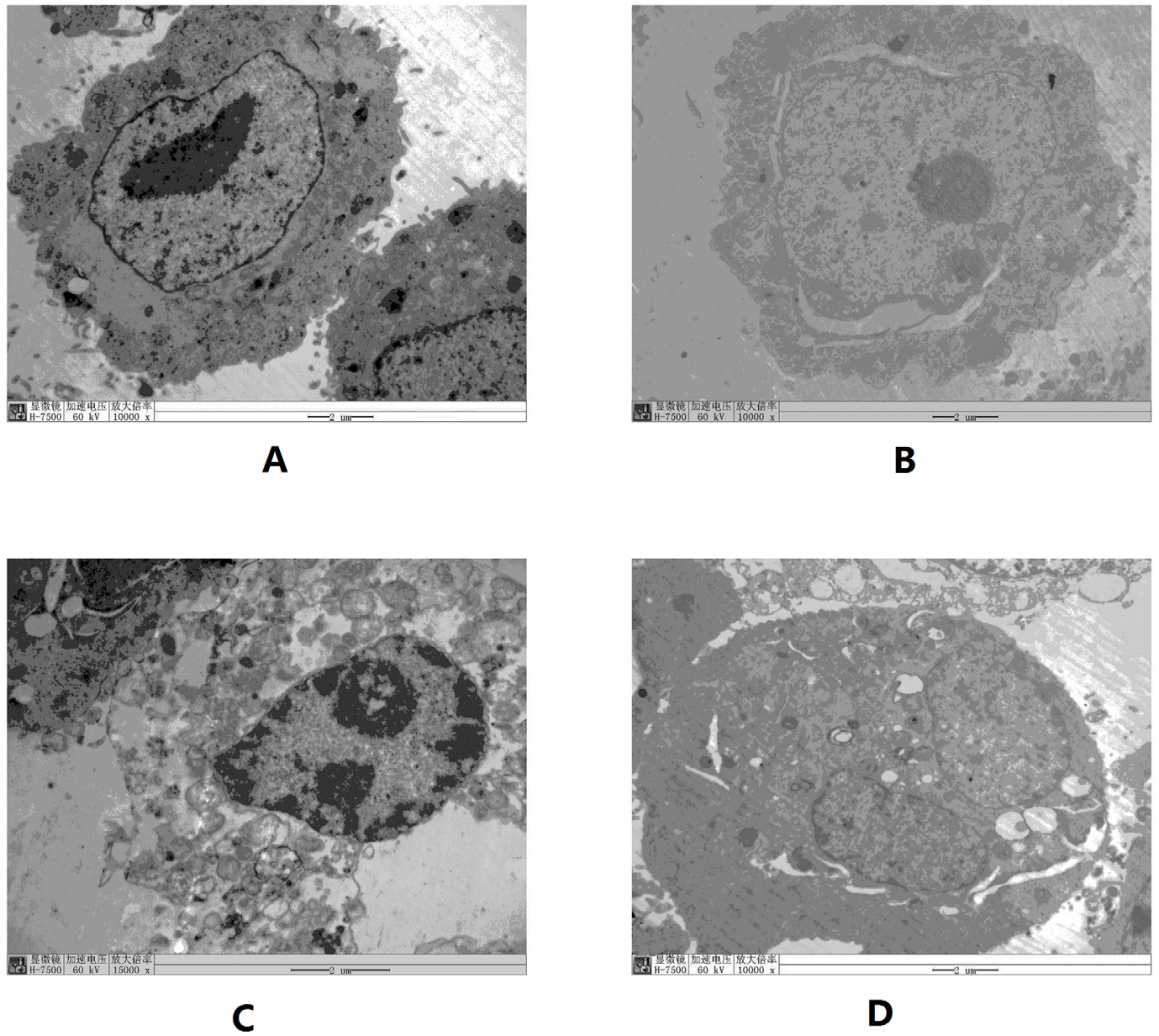
Subcellular structure of infected cells and subcellular localization of virus under electron microscopy. A is normal HBECs (×10000); B is RSV-prolonged infected HBECs in early infection stage, fissure around nucleus is seen (×10000); C is also in early infection stage, expansion of endoplasmic reticulum and a large number of lysosomes in cytoplasm were present (×15000). Both cells in B and C had double nucleoli, which indicated the cell was proliferating rapidly; D is in late infection stage, enlarged fusion cells were present (×10000). Intracellular virus particles indicated by arrows were observed both in nucleus and endoplasmic reticulum.

Under electron microscopy, pathologic changes were not found in normal HBECs (Figure 3A). Mitochondrial swelling, expansion of the endoplasmic reticulum, nuclear fracture, and an abundance of lysosomes were found in RSV-infected HBECs (Figures 3B–D). The infected cells grew rapidly with double nucleoli (Figure 3B), which were present in the former infection stage. Enlarged fusion cells were present in the late infection stage (Figure 3D). Intracellular virus particles were observed in the nucleus and endoplasmic reticulum (Figures 3B and C).

### RSV-infected HBECs induced proliferation and apoptosis of lymphocytes

There are three phases in the cell growth cycle, as follows: pre-synthetic phase (G1 phase); mitotic phase (S phase); and post-synthetic phase (G2 phase). Cells in the G1 and G2 phases are stationary, while cells in the S phase are proliferating. Thus, the proportion of S phase cells may reflect the vitality of cells. Lymphocytes are terminal cells, and may continue proliferating while constantly stimulated by antigens. In general, most lymphocytes are quiescent (G1 phase).

**Figure 4:**
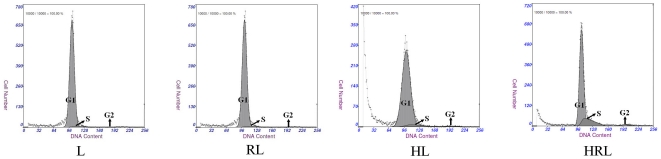
Effect of RSV- infected HBECs on cell cycle of lymphocyte. Flow cytometry analysis showed significant more cells in S phase and less cells in G1 phase in group HRL compared with the other three groups. And even cells in S phase were more in group HL than the rest two groups.

Lymphocytes were co-cultured with RSV-infected HBECs for 24 h, then the cell cycle phases and apoptosis rate of the stained cells were determined. Flow cytometry analysis showed significantly more cells in the S phase and a higher apoptosis rate for group HRL compared with the other three groups. There was a greater number of cells in the S phase in group HL compared with the other two groups. Therefore, RSV-infected HBECs induced significant proliferation and an accelerated apoptosis rate of lymphocytes. Non-infected HBECs may also have a stimulatory effect on lymphocyte proliferation. RSV alone had no effect on proliferation and apoptosis of lymphocytes (Figure 4, and Table 1).

**Table 1:** Effect of RSV-infected HBECs on the cells cycle of lymphocytes.

Group	G0/G1	S	G2/M	Apoptosis rate
L	97.53±1.09	1.38±0.65	1.08±0.72	15.044±5.22
RL	97.30±1.28	1.45±0.89	1.24±0.54	13±3.23
HL	91.08±3.75^*▵^	5.09±1.15^*▵^	2.21±0.84	10.04±4.06
HRL	86.63±3.75^*▵▴^	11.63±6.31^*▵▴^	1.98±1.42	29.88±8.81^*▵▴^

(%, n = 8/group, mean±SEM; *P<0.01 vs L ^▵^P<0.05 vs RL ^▴^P<0.05 vs HL).

#### The release of cytokines by lymphocytes was induced by RSV-infected HBECs

**Figure 5:**
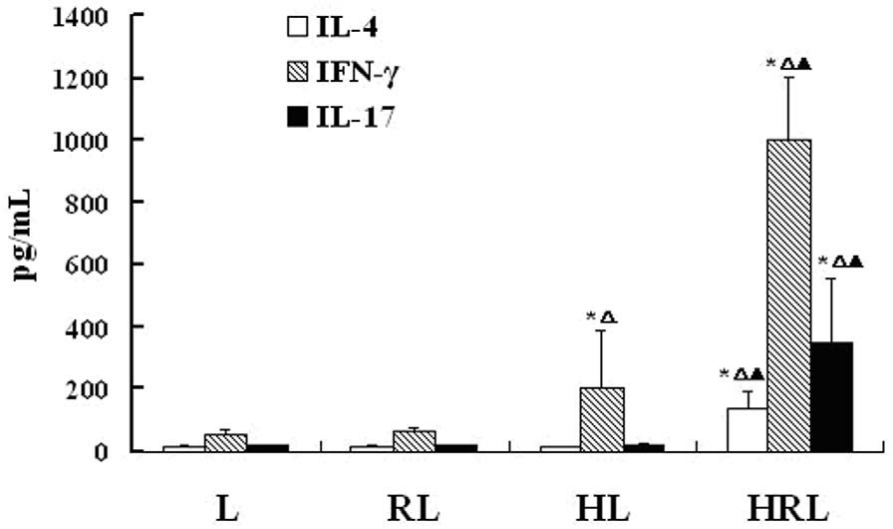
Effect of RSV-infected HBECs on concentrations of IL-4, IFN-γ and IL-17 secreted by lymphocytes (pg/mL, n = 10/group, mean±SEM; *P<0.05 vs. L ^▵^P<0.05 vs. RL ^▴^P<0.05 vs. HL. The levels of IL-4, IL-17 and IFN-γ in group HRL were highest in four groups. The level of IFN-γ in group HL was higher than those of groups L and RL.

Lymphocytes in the resting state released low levels of cytokines. After co-culture with RSV-infected HBECs for 24 h, the release of cytokines from lymphocytes was significantly increased. However, RSV alone and non-infected HBECs had no significant effect on the release of cytokines by lymphocytes. Human IL-4, IFN-γ, and IL-17 concentrations in co-cultured supernatants were measured by ELISA. ELISA results showed that the levels of IL-4, IFN-γ, and IL-17 in group HRL were significantly higher than the other three groups, especially IFN-γ. The level of IFN-γ in group HL was higher than groups L and RL (Figure 5), suggesting that lymphocytes were making an abundance of IL-4 and IL-17, and excessive IFN-γ in response to HBECs infected with RSV.

#### Supernatants from RSV-infected HBECs altered the distribution of Th subsets

**Figure 6:**
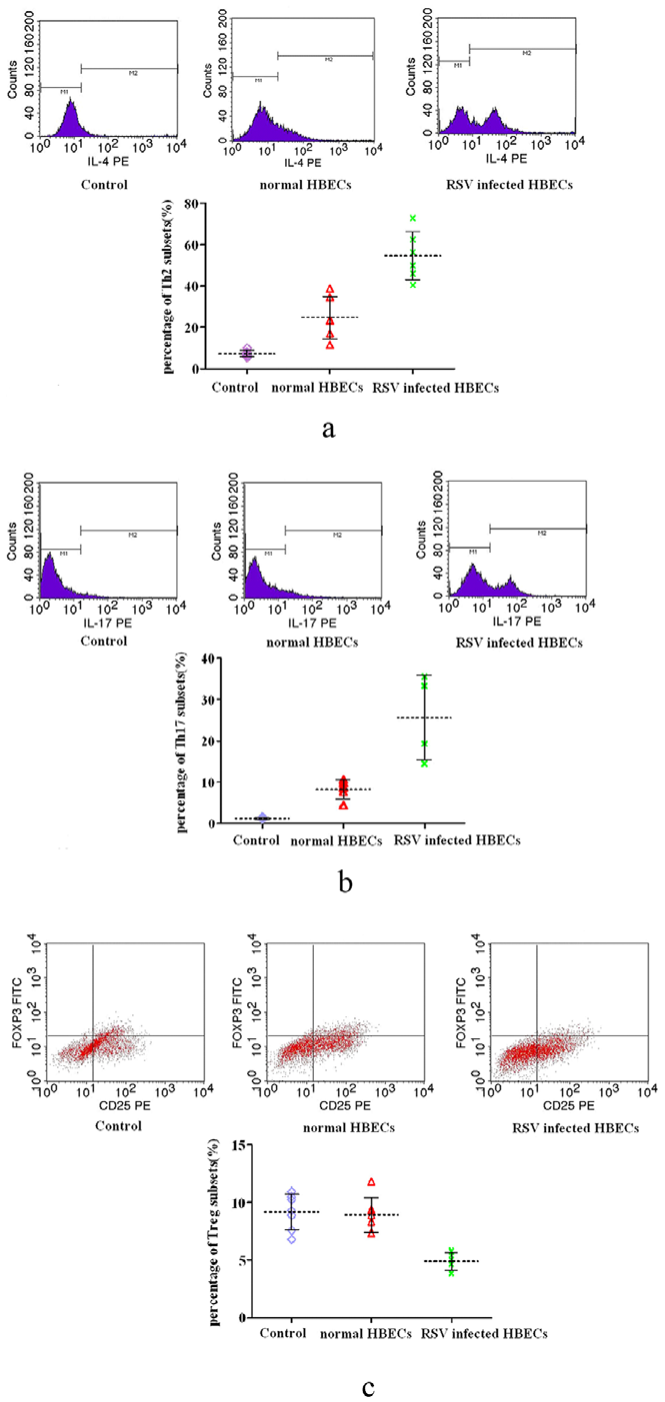
The change of Th subsets in lymphocytes after treatment with supernatants from RSV-infected HBECs. a. The change of Th2 cells in lymphocytes after treatment with supernatants from RSV-infected HBECs. Th2 cells mainly secreted IL-4. Monensin inhibited the secretion of newly produced cytokines in Golgi body. The positive cells in M2 which were conjugated with anti IL-4 were Th2 cells. In Fig 2.6a, the percentage of Th2 cells in lymphocytes co-cultured with RSV-infected HBECs was highest among three groups. The percentage of Th2 cells in lymphocytes co-cultured with normal HBECs was still higher than control. b: The change of Th17 cells in lymphocytes after treatment with supernatants from RSV-infected HBECs. Th17 cells mainly secreted IL-17. The positive cells in M2 which were conjugated with anti IL-17 were Th17 cells. In Fig 2.6b, the percentage of Th17 cells in lymphocytes co-cultured with RSV-infected HBECs was highest among three groups. The percentage of Th2 cells in lymphocytes co-cultured with normal HBECs was still higher than control. c: The change of Treg cells in lymphocytes after treatment with supernatants from RSV-infected HBECs. CD25 was surface marker and Foxp3 was intracellular activating factor of regulatory T cells (Treg). The positive cells in right upper region which were conjugated with anti-CD25 and anti-Foxp3 antibodies were Treg cells. In Fig 2.6c, the percentage of Treg cells in lymphocytes co-cultured with RSV-infected HBECs was lower than the other two groups. There was no difference between control group and normal HBECs group.

**Figure 7:**
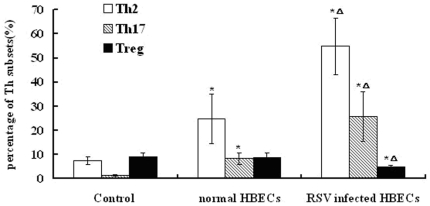
The distribution of Th subsets after treatment with supernatants from RSV-infected HBECs (%, n = 6/group, mean±SEM; *P<0.01 vs. control ^▵^P<0.05 vs. normal HBECs). The percentages of Th2 and Th17 cells in RSV-infected group were higher than the other two groups, while the percentage of Treg was less than the other groups. The percentages of Th2 and Th17 cells in normal HBECs group were higher than control group.

Chemokines and cytokines are crucial in directing the differentiation of Th subsets, and thus serve as attractive targets for intervention [20]. Respiratory epithelial cells, as the principle target for RSV infections, secrete a range of chemokines and cytokines after infection with RSV [16]. In this study, HBECs with prolonged RSV infection might also secrete a range of factors into supernatants which could have an effect on lymphocytes. To test this hypothesis, the distribution of Th subsets was analyzed after individual treatment with complete medium, supernatants from normal HBECs, and supernatants from RSV-infected HBECs for 24 h. The results showed that Th2 and Th17 differentiation was induced by treatment with supernatants from RSV-infected HBECs. However, Treg differentiation was suppressed after treatment with supernatants secreted from RSV-infected HBECs (Figures 6 and 7). Th2 and Th17 differentiation was stimulated to some extent by treatment with supernatants from normal HBECs.

## Discussion

In most previous studies, acute RSV infection models have been established. The infected cells underwent apoptosis progressively in hours or days and could not continue to split and passage. However, studies [21] in recent years showed that chronic infection with RSV could aggravate the frequency and severity of acute exacerbation in some respiratory diseases such as COPD. It has been observed in animal experiments [22], [23] that RSV is able to escape immune clearance from the host and be persistently latent in lung tissues; therefore, the immune system in infected hosts might be stimulated or stressed persistently. In respiratory tracts of chronically infected animals, inflammatory mediators are released and inflammatory cells are recruited. Using a macrophage model, Guerrero-Plata et al. [24] reported that persistent/prolonged RSV infections stimulated macrophages to oversecrete pro-inflammatory cytokines, and promoted the antigen presenting function, suggesting that persistent RSV infections may be responsible for increased susceptibility to respiratory inflammatory diseases, such as asthma. Airway epithelial cells have the potential for presenting antigens, releasing inflammatory mediators, and activating the immune response, and at the same time are main targets of RSV infection. Thus, we speculate that persistent/prolonged RSV infections induce airway inflammation or aberrant immune reactions through changing functions of airway epithelial cells. Therefore, a prolonged infection model with RSV in HBECs was established in this study. The main characteristic of this model was that infected cells had the ability to proliferate and passage, and also maintained an active biological state. In the early infection stage, the cells proliferated actively. In the late infection stage, enlarged fusion cells were present, which were considered to be typical lesions of RSV infections.

When subjected to invasion of external pathogens (bacteria and viruses), the human body will initiate immune protection against pathogens. Exogenous microbial antigens are taken up by APCs and presented to lymphocytes, which trigger immune responses. Proliferation and activation of lymphocytes are in response to Th cell differentiation and the release of large amounts of cytokines. Cytokines also promote source lymphocytes and other immune cells to further differentiate and proliferate, and subsequently generate a wide range of immune effector cells [25]. In addition, lymphocyte apoptosis, which is also one of the host immune responses, is an antagonistic mechanism against excessive inflammatory responses [26]. In that case, apoptosis of recruited lymphocytes is induced and minimizes the damage of inflammatory tissues [27]. Our results showed that after co-culture with HBECs with prolonged RSV infection, the proliferation and apoptosis of lymphocytes were enhanced as well as excessive secretion of IL-4, IFN-γ, and IL-17, which may suggest that lymphocytes had received the stimulation of external antigen and subsequently activated the immune response. Only RSV had no effect on proliferation, apoptosis and cytokine release of lymphocytes. Thus, it was considered that RSV alone could not activate lymphocytes. The antigen signals which triggered activation of lymphocytes might be processed and synthesized by HBECs which absorbed RSV pathogenic proteins, thereby activating the function of lymphocytes in immune responses. Non-infected HBECs also induced low-level lymphocyte proliferation and secretion of IFN-γ. In addition to RSV, there must exist weak antigen signals in the external environment which promotes HBECs to activate lymphocytes. In addition, IFN-γ might serve as a sensitive indicator to environmental changes, and even a weak signal could induce the expression and release of IFN-γ [28]; however, HBECs which are only infected with RSV, can fully activate lymphocytes.

As the first barrier to the external environment in the respiratory tract, airway epithelial cells play an important role in maintaining homeostasis of the local microenvironment [19]. Some chemical signals or exogenous stimuli in the local micro-environment could cause adaptive functional changes of epithelial cells [29]. In the co-culture system of our experiments, HBECs activated lymphocytes after prolonged infection with RSV. One of the possible mechanisms is that infected HBECs, after contacting lymphocytes, directly presented activating signals to lymphocytes. The activation and differentiation of T cells require a specific antigen peptide-MHCII molecule complex (first signal) which is processed and synthesized by APCs. In addition to the first signal, B7-1 (CD80) and B7-2 (CD86) and other co-stimulatory molecules combined with CD28 expressed by T cells are the second signal to activate T cells. It has been shown that BECs in stress or pathologic conditions can express MHCI and MHCII molecules, then mediate T cell migration [30], [31]. Therefore, the BECs are considered to have potential antigen-presenting functions. In animal models of asthma, HLA-DR and HSP70, two types of molecules with antigenic characteristics, are over-expressed in airway epithelium [32]. Papi et al. [33] reported that B7-1 and B7-2 co-stimulatory molecules are expressed in lung cancer cell lines and HBECs after infection with rhinovirus. Thus, it is well-accepted that airway epithelial cells are the first line of defense that have early contact with pathogenic antigen and initiate specific immune responses. In this study, HBECs absorbed RSV pathogenic proteins first, then processed and synthesized antigen-presenting complex as the first signal, as well as expressed co-stimulatory molecules as the second signal, which comprised the activation signals of lymphocytes.

The common precursor cells of Th subsets are Th0 cells. According to various media, the type and concentration of antigens and type of APCs, Th0 cells respectively differentiate to Th1, Th2, Th17, and Treg subsets, which is referred to as Th subset drift, and also known as functional polarization. Th subset drift is also a performance part of lymphocyte activation. Among many influential factors, the cytokine microenvironment, in which Th0 cells contact APCs, is important in regulating Th cell differentiation. For example, IL-12 and IFN-γ promote Th0 cells to differentiate to Th1 cells [34], [35], and IL-4 promotes Th0 cells to differentiate to Th2 cells.

Our results showed that HBECs with prolonged RSV infection induced lymphocytes to secrete large quantities of IFN-γ, IL-4 and IL-17, which could be the result of an altered pattern of Th subset differentiation after stimulation by RSV-infected epithelial cells. Moreover, we provide further evidence that abnormal differentiation of Th cells was observed by secreted supernatants from RSV-infected HBECs, which could induce the differentiation of Th2 and Th17 lymphocytes, and suppress the differentiation of Treg cells. Because Th subset drift is mainly affected by the micro-environment, cytokines or other factors oversecreted by RSV-infected epithelial cells might cause a change in the microenvironment, which could be an original cause that indirectly contributes to the pathologic differentiations of Th subsets. The immune responses of epithelia to infection and antigen exposure also involve the release of chemokines and cytokines into the submucosa, and thus initiate an inflammatory reaction. Chemokines belong to a family of chemotactic cytokines that have been shown to play an integral role in a variety of biological activities. Each chemokine has the ability to chemoattract a particular subset of inflammatory cells. Therefore, one can imagine that the chemokine profile that follows a particular stimulus will dictate the subsequent inflammatory response. RSV predominantly infects airway epithelial cells. *In vitro*, RSV infection of human airway epithelial cells has been shown to release a wide range of chemokines including intercellular adhesion molecule (ICAM)-1 [36], IL-6 [19], IL-8 [37], and regulation upon activation normal T cell-expressed and secreted (RANTES) [38]. Those chemokines have been demonstrated to initiate neutrophil, Th cell and eosinophil recruitment and differentiation, as well as enhance the expression of inflammatory transcription factor NF-κB [39]. However, the profile of chemokines secreted by HBECs infected with RSV, which can affect Th subsets differentiation needs to be further analyzed.

RSV infection may increase the risk of asthma morbidity, but the underlying mechanism remains unknown. In allergic asthma, a critical event is the activation of Th cells, leading to a predominance of Th2 cytokines over Th1 cytokines [40]. A recent study suggests that Th1, as well as Th2 cytokines, may promote asthma [41]. Also, T cell subsets, in addition to Th1 and Th2, have been identified in asthma, including Treg and Th17 cells [42]. In allergic disease, such as asthma, Treg can suppress Th2 responses to allergen [43] and Th17 cells play a role in promoting inflammatory response [44]. The pathologic changes induced by HBECs with prolonged RSV infection, including the activation of lymphocytes, Th2, Th17 proliferation, and Treg cell suppression observed in our study were consistent with the reported immune system changes in asthma. Our data suggest that susceptibility to asthma after RSV infection would be related to lymphocyte activation and immune imbalance caused by RSV. Bronchial epithelial cells play a key role by presenting activation signals and changing the secretory pattern after RSV infection.

For flow cytometry analysis, the data of Th1 cells was not included herein. In the past it was thought that Th1 cells and IFN-γ play a protective role in asthma. A recent study has suggested that Th1, as well as Th2 cytokines, may promote asthma, and IFN-γ can also promote the development of inflammation. Thus, the role of Th1 cells and IFN-γ in asthma, especially in RSV-induced asthma, remains unclear. At the same time, the value of the Th1 subset determined by flow cytometry was unstable and a definite conclusion was difficult to obtain. As is known, IFN-γ was significantly increased, especially in the viral infection, but the role in asthma was not as clear as IL-4 and IL-17 until now. Therefore, the increased level of IFN-γ examined by ELISA suggested that lymphocytes were activated by antigen-presenting signal containing viral antigen. Increased proportions of Th2 and Th17 subsets tested by flow cytometry suggested that RSV infection is closely related to asthma.

## References

[1] Simoes EA (1999). Respiratory syncytial virus infection.. Lancet.

[2] Webb MS, Henry RL, Milner AD, Stokes GM, Swarbrick AS (1985). Continuing respiratory problems three and a half years after acute viral bronchiolitis.. Arch Dis Child.

[3] Hall CB, Long CE, Schnabel KC (2001). Respiratory syncytial virus infections in previously healthy working adults.. Clin Infect Dis.

[4] Hall CB (2001). Respiratory syncytial virus and parainfluenza virus.. N Engl J Med.

[5] Busse WW (2000). Mechanisms and advances in allergic diseases.. J Allergy Clin Immunol.

[6] Gern JE, Busse WW (2000). The role of viral infections in the natural history of asthma.. J Allergy Clin Immunol.

[7] Hu C, Wedde-Beer K, Auais A, Rodriguez MM, Piedimonte G (2002). Nerve growth factor and nerve growth factor receptors in respiratory syncytial virus-infected lungs.. Am J Physiol Lung Cell Mol Physiol.

[8] Kawano Y, Noma T (1995). Modulation of mite antigen-induced immune responses by lecithin-bound iodine in peripheral blood lymphocytes from patients with bronchial asthma.. Br J Pharmacol.

[9] Mosmann TR, Cherwinski H, Bond MW, Giedlin MA, Coffman RL (1986). Two types of murine helper T cell clone. I. Definition according to profiles of lymphokine activities and secreted proteins.. J Immunol.

[10] Paul WE, Seder RA (1994). Lyphocyte responses and cytokines.. Cell.

[11] Nakajima H, Hirose K (2010). Role of IL-23 and Th17 Cells in airway inflammation in asthma.. Immune Netw.

[12] Ziegler SF (2006). FoxP3: of mice and men.. Annu Rev Immunol.

[13] Kim CH (2006). Migration and function of FoxP3+ regulatory T cells in the hematolymphoid system.. Exp Hematol.

[14] Yoon JS, Kim HH, Lee Y, Lee JS (2007). Cytokine induction by respiratory syncytial virus and adenovirus in bronchial epithelial cells.. Pediatr Pulmonol.

[15] Holgate ST (2008). The airway epithelium is central to the pathogenesis of asthma.. Allergol Int.

[16] Hacking D, Hull J (2002). Respiratory syncytial virus-viral biology and the host response.. J Infect.

[17] Yin S, Sun S, Yang S, Shang Y, Cai X (2010). Self-assembly of virus-like particles of porcine circovirus type 2 capsid protein from Escherichia coli.. Virol J.

[18] Boyum A (1968). Isolation of mononuclear cells and granulocytes from human blood.. Scand J Clin Lab Invest.

[19] Tristram DA, Hicks W, Hard R (1998). Respiratory syncytial virus and human bronchial epithelium.. Arch Otolaryngol Head Neck Surg.

[20] Openshaw PJ, Tregoning JS (2005). Immune responses and disease enhancement during respiratory syncytial virus infection.. Clin Microbiol Rev.

[21] Seemungal T, Harper-Owen R, Bhowmik A, Moric I, Sanderson G (2001). Respiratory viruses, symptoms, and inflammatory markers in acute exacerbations and stable chronic obstructive pulmonary disease.. Am J Respir Crit Care Med.

[22] Mejías A, Chávez-Bueno S, Gómez AM, Somers C, Estripeaut D (2008). Respiratory syncytial virus persistence: evidence in the mouse model.. Pediatr Infect Dis J.

[23] Schwarze J, O'Donnell DR, Rohwedder A, Openshaw PJ (2004). Latency and persistence of respiratory syncytial virus despite T cell immunity.. Am J Respir Crit Care Med.

[24] Guerrero-Plata A, Ortega E, Ortiz-Navarrete V, Gómez B (2004). Antigen presentation by a macrophage-like cell line persistently infected with respiratory syncytial virus.. Virus Res.

[25] Holub M (1967). The lymphocyte and the immune response.. Mod Trends Immunol.

[26] Shi X, LeCapitaine NJ, Rudner XL, Ruan S, Shellito JE (2009). Lymphocyte apoptosis in murine Pneumocystis pneumonia.. Respir Res.

[27] Strasser A, Pelleginni M (2004). T-lymphocyte death during shutdown of an immune response.. Trends Immunol.

[28] Nakagome K, Okunishi K, Imamura M, Harada H, Matsumoto T (2009). IFN-gamma attenuates antigen-induced overall immune response in the airway as a Th1-type immune regulatory cytokine.. J Immunol.

[29] Wark PA, Grissell T, Davies B, See H, Gibson PG (2009). Diversity in the bronchial epithelial cell response to infection with different rhinovirus strains.. Respirology.

[30] Kalb TH, Chuang MT, Marom Z, Mayer I (1991). Evidence for accessory cell function by class IIMHC antigen-expressing airway epithelial cells.. Am Respir Cell Mol Biol.

[31] Kurosawa S, Myers AC, Chen L, Wang S, Ni J (2003). Expression of the costimulatory molecule B7-H2 by human airway epithelial cells.. Am Respir Cell Mol Biol.

[32] Bertorelli G, Bocchino V, Zhou X, Chentta A, Del Donno M (1998). Heat shock protein 70 upregulation is related to HLA-DR expression in bronchial asthma.. Clin Exp Allergy.

[33] Papi A, Stanciu LA, Papadopoulos NG, Teran IM, Holgate ST (2000). Rhinovirus infection induces major histocompatibility complex class I and costimulatory molecule upregulation on respiratory epithelial cells.. J Infect Dis.

[34] Padigel UM, Perrin PJ, Farrell JP (2001). The development of a Th1 type response and resistance to Leishmania major infection in the absence of CD40-CD40L costimulation.. J Immunol.

[35] Yoshimura C, Nomura S, Kanazawa S, Kuwana M, Yamaguchi K (2002). Comparison of interleukin-12 with lung cancer and malignant lymphoma undergoing autologous peripheral blood stem cell transplantation.. Cancer Res Clin Oncol.

[36] Patel JA, Kunimoto M, Sim TC, Garofalo R, Eliott T (1995). Interleukin-1a mediates the enhanced expression of intracellular adhesion molecule-1 in pulmonary epithelial cells infected with respiratory syncytial virus.. Am J Respir Cell Mol Biol.

[37] Noah TL, Henderson FW, Wortman IA, Devlin RB, Handy J (1995). Nasal cytokine production in viral acute upper respiratory infection of childhood.. J Infect Dis.

[38] Becker S, Reed W, Henderson FW, Noah TL (1997). RSV infection of human airway epithelial cells causes production of the beta-chemokine RANTES.. Am J Physiol.

[39] Jamaluddin M, Choudhary S, Wang S, Casola A, Huda R (2005). Respiratory syncytial virus-inducible BCL-3 expression antagonizes the STAT/IRF and NF-kB signaling pathways by inducing histone deacetylase 1 recruitment to the interleukin-8 promoter.. J Virol.

[40] Finn PW, Bigby TD (2009). Innate immunity and asthma.. Proc Am Thorac Soc.

[41] Castro M, Chaplin DD, Walter MJ, Holtzman MJ (2000). Could asthma be worsened by stimulating the T-helper type 1 immune response.. Am J Respir Cell Mol Biol.

[42] Ouyang W, Kolls JK, Zheng Y (2008). The biological functions of T helper 17 cell effector cytokines in inflammation.. Immunity.

[43] Larche M (2007). Regulatory T cells in allergy and asthma.. Chest.

[44] Zhou Liang, Littman DR (2009). Transcriptional regulatory networks in Th17 cell differentiation.. Curr Opin Immunol.

